# Overcoming the Fermi-Level Pinning Effect in the Nanoscale Metal and Silicon Interface

**DOI:** 10.3390/nano13152193

**Published:** 2023-07-28

**Authors:** Zih-Chun Su, Ching-Fuh Lin

**Affiliations:** 1 Graduate Institute of Photonics and Optoelectronics, National Taiwan University, Taipei 10617, Taiwan; f06941006@ntu.edu.tw; 2 Graduate Institute of Electronics Engineering, National Taiwan University, Taipei 10617, Taiwan; 3 Department of Electrical Engineering, National Taiwan University, Taipei 10617, Taiwan

**Keywords:** ultra-broadband infrared photon detection technique, Schottky devices, Fermi-level pinning, interface passivation

## Abstract

Silicon-based photodetectors are attractive as low-cost and environmentally friendly optical sensors. Also, their compatibility with complementary metal-oxide-semiconductor (CMOS) technology is advantageous for the development of silicon photonics systems. However, extending optical responsivity of silicon-based photodetectors to the mid-infrared (mid-IR) wavelength range remains challenging. In developing mid-IR infrared Schottky detectors, nanoscale metals are critical. Nonetheless, one key factor is the Fermi-level pinning effect at the metal/silicon interface and the presence of metal-induced gap states (MIGS). Here, we demonstrate the utilization of the passivated surface layer on semiconductor materials as an insulating material in metal-insulator-semiconductor (MIS) contacts to mitigate the Fermi-level pinning effect. The removal of Fermi-level pinning effectively reduces the Schottky barrier height by 12.5% to 16%. The demonstrated devices exhibit a high responsivity of up to 234 μA/W at a wavelength of 2 μm, 48.2 μA/W at 3 μm, and 1.75 μA/W at 6 μm. The corresponding detectivities at 2 and 3 μm are 1.17 × 10^8^ cm Hz^1/2^ W^−1^ and 2.41 × 10^7^ cm Hz^1/2^ W^−1^, respectively. The expanded sensing wavelength range contributes to the application development of future silicon photonics integration platforms.

## 1. Introduction

With the development of the Internet of Things (IoT) and smart living, our lives are increasingly convenient with the help of artificial intelligence and various sensors. As one of the most important sensing technologies, photodetectors that can accurately convert incident light into electrical signals are receiving more attention. Broadband photodetectors, ranging from ultraviolet to mid-infrared, have been widely applied in spectroscopic analysis, environmental monitoring, communication, imaging, and display. Traditional indicators used to evaluate photodetectors include stability, signal-to-noise ratio, sensitivity, speed, and selectivity. However, with the rapid advancement of miniaturized smart devices, new types of photodetectors require additional compatibility with integrated circuits to provide higher-level computational analysis functions, such as logic analysis.

High-performance broadband photodetectors predominantly utilize costly III–V materials [1,2,3] or two-dimensional materials [4]. For instance, in the case of III–V materials like InAs/InAsSb superlattices, these detectors consist of a superlattice contact layer and an absorbing layer with an intermediate electron-blocking layer [1]. The detection wavelength range is tailored by adjusting the periodicity of the superlattice in the absorbing layer. However, integrating III–V materials with other substances presents challenges due to lattice mismatch, leading to the formation of dislocations that significantly degrade device performance [5,6,7]. Consequently, the materials employed in such detectors require specialized epitaxial techniques or additional electronic components for integration with silicon ICs, thereby complicating the direct realization of miniaturized smart devices and limiting their suitability to conventional applications. In contrast, silicon IC-compatible photodetectors enable the fabrication of silicon electronic devices and optical components on a single chip. This facilitates the creation of monolithic photon-electron systems with inherent capabilities for logic computation, storage, and interconnectivity, which assume significant importance in the present scenario.

Based on mature silicon-based processing technology, the material selection for silicon-based photodetectors has greatly benefited from p-type or n-type doping. The p-i-n type silicon photodetectors [5,6,7] are widely used for visible light applications, including optical imaging and spectroscopic analysis. Additionally, metal-silicon and metal-oxide-silicon photodiodes offer improved electrical properties. However, silicon materials also exhibit limitations in terms of photosensitivity. The indirect bandgap of silicon makes it an incomplete optoelectronic material, as its material bandgap of 1.1 eV leads to reduce absorption beyond 800 nm, with a cutoff wavelength of around 1100 nm [8,9]. Consequently, silicon is not considered an ideal optoelectronic material beyond the visible light range. Furthermore, the non-direct bandgap characteristic of silicon results in decreased photodetection performance and introduces thermal noise. Overcoming these challenges and striving for higher-performance silicon-compatible photodetectors remains of utmost importance.

In recent years, the development of hot carrier technology at metal/silicon interface has attracted considerable attention and brought new concepts and approaches to silicon-based infrared sensing [10,11,12]. Material selection plays an important role in achieving photoinduced hot carriers that are able to absorb infrared light on the silicon photonics system. Metal materials, due to their zero bandgap properties, have the potential to achieve broadband photon absorption [13,14,15,16] and are promising for infrared sensing. The material selection plays an important role in achieving photoinduced hot carriers that are able to absorb infrared light on the silicon photonics system. Metal materials, due to their zero bandgap properties, have the potential to achieve broadband photon absorption [13,14,15,16] and are promising for infrared sensing. Considering the special hot-carrier behaviors in nanoscale metals, increasing research has shown interest in developing infrared Schottky detectors using metals with nanometer features. In 2017, Zhiyang et al. proposed an efficient and low-cost plasmonic hot electron NIR (1200–1475 nm) photodetector based on an Au nanoparticle [14]. In 2020, Yusheng et al. demonstrated that the enhanced responsivity is related to the location of the field enhancement, the responsivity of the nanoscale-Au film device was 1.8 mA/W at 1310 nm [15]. These results already break the detection cutoff wavelength (1.1 μm) of the silicon material.

Nanoscale metals’ potential to achieve infrared photodetector have already been demonstrated. However, in metal/silicon interface photodetectors, the presence of Fermi-level pinning limits the barrier height and further restricts the detectable wavelength range of nanoscale-metal/silicon interface infrared photodetectors. Since Fermi levels of two materials must align with each other at the interface, there exist gap states that decay deeper into the semiconductor, known as metal-induced gap states (MIGS) responsible for pinning of the surface energy state regardless of the metal used, namely the Fermi-level pinning. The dominant characteristic of these interface states changes from acceptor-like to donor-like, resulting in charge transfer across the interface and the formation of a dipole that tends to align the band edges at the zero-charge level, effectively pinning the metal Fermi level at the center of the semiconductor bandgap [17,18,19,20,21]. Consequently, regardless of the metal used, there exists a barrier height of about 1/2 of the semiconductor bandgap. It is known that inserting an insulating layer between the semiconductor and the metal can reduce the effect of Fermi-level pinning [22,23,24,25,26]. The purpose of the insulating layer is to weaken the penetration of metal wave functions into the semiconductor, reducing MIGS and, thus, reducing the pinning effect. However, the presence of the insulating layer also introduces high tunneling resistance, requiring a trade-off between reducing pinning and increasing tunneling in practical implementations.

Until now, most research efforts on Fermi-level pinning have focused on optimizing electrode contacts in solar cells [27,28,29] and gate characteristics in field-effect transistors [30,31,32,33,34]. Research on Fermi-level pinning in the hot carrier detection devices has mainly focused on enhancing the photoelectric response in the visible to near-infrared wavelength range [14,15,16]. There has been relatively less discussion on using metal-insulator-semiconductor (MIS) structures to alleviate Fermi-level pinning, reduce the effective barrier height, and improve the device response in the infrared wavelength range. In this work, we experimentally demonstrate that the barrier height variation at the Schottky junction strongly influences the detection capability of infrared photodetectors. By striking a balance between reducing pinning and increasing tunneling, the metal-silicon dioxide-silicon photodetector exhibits a response rate of 2.34 × 10^2^ μA/W at 2 μm and 1.75 μA/W at 6 μm.

## 2. Materials and Methods

### 2.1. Device Fabrication

**Figure 1:**
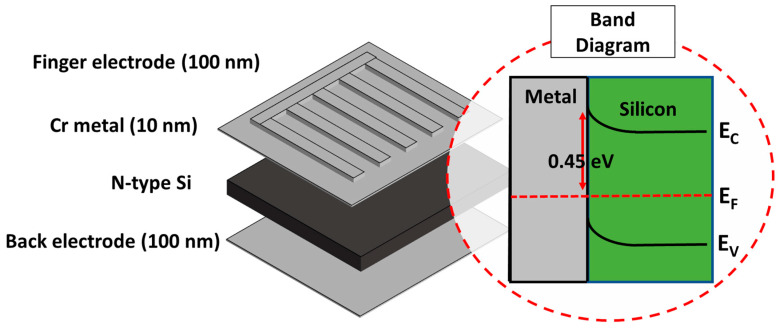
Component diagram and ideal band diagram.

In the fabrication experiment, a (100) n-type silicon wafer with a resistivity of 2–7 Ω-cm, a thickness of 650 μm, and a phosphorus doping concentration of 7 × 10^14^ cm^−3^ was used. Initially, organic materials and the surface oxide layer were removed using organic solvents and buffered oxide etchant (BOE), respectively. Then, the cleaned wafer was loaded into an electron beam evaporation system (e-gun) to deposit a metal film. To form the Schottky contact, a 10 nm thick chromium layer was deposited on the substrate, followed by the deposition of a finger-shaped electrode with a thickness of 90 nm, resulting in a total thickness of 100 nm in the region with the finger-shaped electrode. Finally, a conductive layer was deposited on the backside as the back electrode to complete the device fabrication. The complete device structure is shown in Figure 1. After the formation of the chromium-silicon Schottky contact, the Fermi levels of the metal and the semiconductor align, creating a barrier at the interface. Using the work function of the metal and the electron affinity of the semiconductor, the ideal chromium/silicon interface has a barrier height of 0.45 eV. However, due to the presence of Fermi-level pinning, the Fermi level after junction formation is mostly pinned near the middle of the silicon bandgap. This can result in a high Schottky barrier, hindering carrier transport and reducing the detected photocurrent. Since silicon can form a silicon dioxide protective layer on the surface by oxidation in a high-temperature environment or in suitable chemical solutions, which is hard and insulated, it is possible to directly oxidize a silicon dioxide insulating layer on the silicon substrate surface through thermal oxidation or chemical oxidation. The modification of the interface dielectric of the silicon semiconductor was tested in next chapter to see whether it could reduce the Fermi-level pinning effect and further enhance the photoelectric response.

### 2.2. Device Characterization

The thermionic emission theory was utilized to fit and analyze the voltage-current characteristic curves of Schottky devices in many research [35,36,37,38]. There are many important physical parameters at the Schottky interface in thermionic emission theory, including the Schottky barrier height, series resistance, and ideality factor [38], which is able to affect the response. In order to discuss the relationship between optical response and the barrier height, series resistance and ideality factor, we need a more accurate method of estimation. The methods of “directly fitting the characteristic curves” and “approximate estimation methods ignoring the series resistance” that may have estimation errors were excluded. Instead, we adopt the approach of “introducing additional adjustable physical variables and validating through repeated fitting” to find the most accurate fitting results. If the adjusted values are consistent with the variations in the corresponding analysis factors, it indicates the accuracy of the analysis results. The fitting equation of the thermionic emission theory for Schottky devices is shown below [38]:

(1) $$I={\mathrm{AA}}^{*}T^{2}\exp(\frac{-q\Phi_{B}}{\mathrm{kT}})[\exp(\frac{V-{\mathrm{IR}}_{s}}{\mathrm{nkT}/q})-1]$$

In Equation (1), V and I represent the applied bias voltage and the measured current of the device, respectively. A, A*, k, and q are known parameters representing the area of device, Richardson constant, Boltzmann constant, and electron charge, respectively. The analysis parameters include R_s_ (series resistance), Φ_B_ (barrier height), T (temperature), and n (ideality factor). For Schottky devices, an equivalent circuit with a series resistance can be utilized. Introducing an additional controllable series resistance as an experimental variable facilitates easy modification and adjustment of the circuit for testing purposes. The measurement system for the device’s external resistance involves direct series connection with the device, while the IV characteristic curves are recorded using a Keithley 2400 source meter. The additive nature of resistance values in series allows the fitting result of the series resistance to validate the overall reference capability of the complete set of fitting parameters. By evaluating the numerical error rate between the fitted and actual added resistance, we can determine the most reliable parameter combination among the different fitting results.

## 3. Results

The metal/silicon interface, as a thermal carrier technology, is primarily determined by three key processes in its optoelectronic response: light absorption, hot carrier generation, and hot carrier transport and collection. When the metal/silicon interface is excited by light, plasmonic excitations are generated and decay, transferring energy to hot electrons. Due to the relaxation process of hot carriers, the hot electrons dissipate energy in the form of heat. However, through the formation of Schottky contacts between metal nanostructures and semiconductors, hot electrons with enough energy are able to be injected into the semiconductor and collected before their energy is lost.

**Figure 2:**
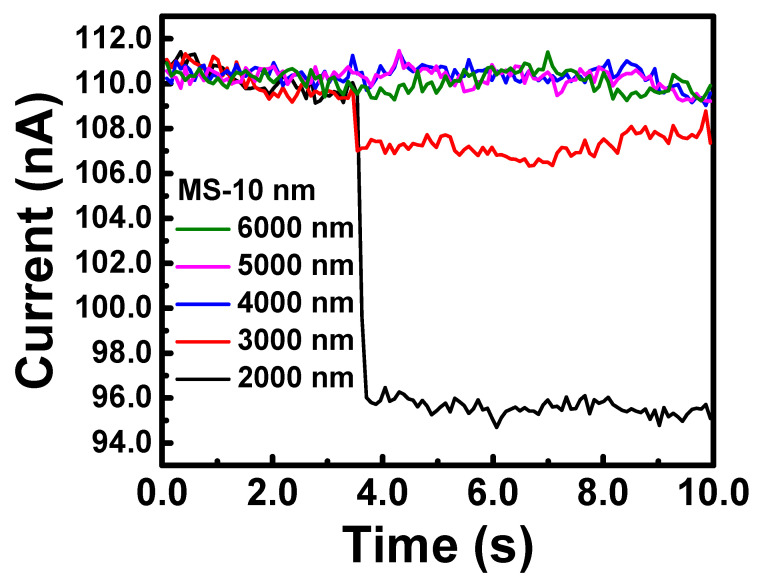
The response measurement of 10 nm metal-semiconductor device.

In this study, we focus on discussing how the Schottky barrier height affects the injection efficiency of hot carriers. Since the parameters of the metal material and fabrication are fixed, and to verify the optical response of the device in the infrared wavelength range, a stable and adjustable infrared light source is required. A monochromator (CM110) controlled by LabVIEW software was used to control the detection wavelength for device measurements. Broadband infrared light ranging from 1.5 to 20 μm was separated into single-wavelength monochromatic light from 2 to 6 μm. The switching of the light signal was controlled by a chopper operating with a 10 s switching period. By analyzing the variation in the current levels of the device with and without infrared light illumination, the optical response of the device could be calculated. As a control group for the experiment, we fabricated devices with an active layer metal thickness of 10 nm and measured their response and absorption spectra. Figure 2 shows the variation in current levels for the device in the wavelength range of 2 to 6 μm.

**Figure 3:**
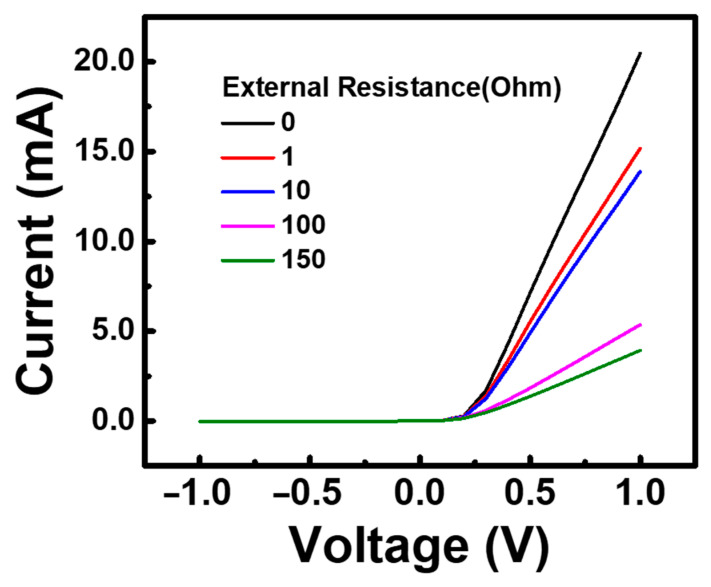
Measurement of characteristic curves with different resistance.

The data of the infrared light source measurements for the device in Figure 2 were quantitatively analyzed. First, the response current was calculated by subtracting the current level without light illumination from the current level during light illumination. The device with an active layer metal thickness of 10 nm with a response current of 14 nA at a wavelength of 2 μm. Although the response was smaller at the 3 μm wavelength, there was still a response current of 2.9 nA. Next, the dark current variation in the barrier height was further measured and analyzed. In order to discuss the relationship between optical response and the barrier height, the current-voltage characteristic curves for different external resistances ranging from 0 to 150 Ω were measured, and the results are shown in Figure 3. For the Schottky barrier device, the circuit can be equivalently represented as a circuit with a series resistance. Therefore, if the fitting result is accurate, the value of the additional series resistance will only be reflected in the change of the fitting resistance value. Equation (1) was used in this analysis. Table 1 records the results of the analysis for R_s_, n, barrier height, and temperature. To estimate the error rate, ΔR_s_ is defined as the difference between the fitting results with and without an external resistance. The relative error is defined as the difference between ΔR_s_ and Rex divided by Rex. Since the error rate of the resistance meter used is 5%, a 5% standard was also adopted when evaluating the fitting results. Under the evaluation standard of an error rate < 5%, the series resistance, ideality factor, and barrier height of the chromium metal/n-Si device we fabricated were determined to be 29.9 Ω, 1.49, and 0.56 eV, respectively. From these results, it can be observed that the device exhibits a significant Fermi-level pinning effect, with a value very close to the midpoint of the silicon bandgap (0.55 eV), showing a deviation of 0.11 eV from the ideal chromium/n-Si barrier of 0.45 eV. If the barrier can be further reduced, there is a chance of measuring signals at longer wavelengths.

**Figure 4:**
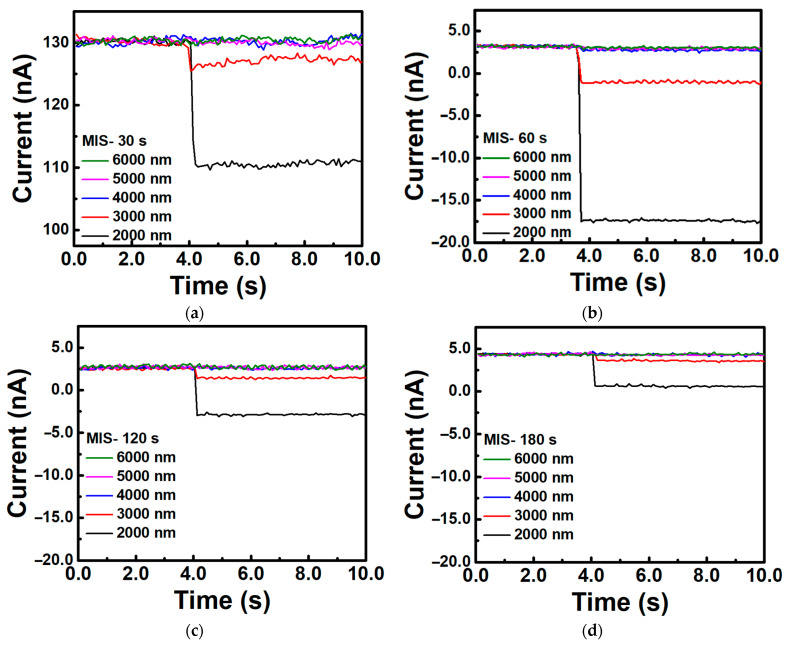
Infrared light response of Cr/SiO_2_/n-Si devices with different oxidation times. (**a**) 30 s, (**b**) 60 s, (**c**) 120 s, (**d**) 180 s.

To reduce the Fermi-level pinning effect, we incorporated an oxidation process on the surface of the fabrication. A piranha solution of sulfuric acid and hydrogen peroxide was utilized to generate a uniform and stable oxide layer on the silicon substrate surface, while removing organic materials. This oxide layer provides good insulation and chemical stability, effectively suppressing background noise in the Schottky barrier device. The oxidation times were set at 30, 60, 120, and 180 s, while the metal thickness was chosen to be 10 nm. Figure 4a–d show the variation in current levels with time at wavelengths of 2 to 6 μm for devices subjected to oxidation times of 30, 60, 120, and 180 s, respectively. For comparison, we also quantitatively analyzed the data of the infrared light source measurements for each parameterized device in Figure 4 and compiled the results in Table 2.

## 4. Discussion

**Figure 5:**
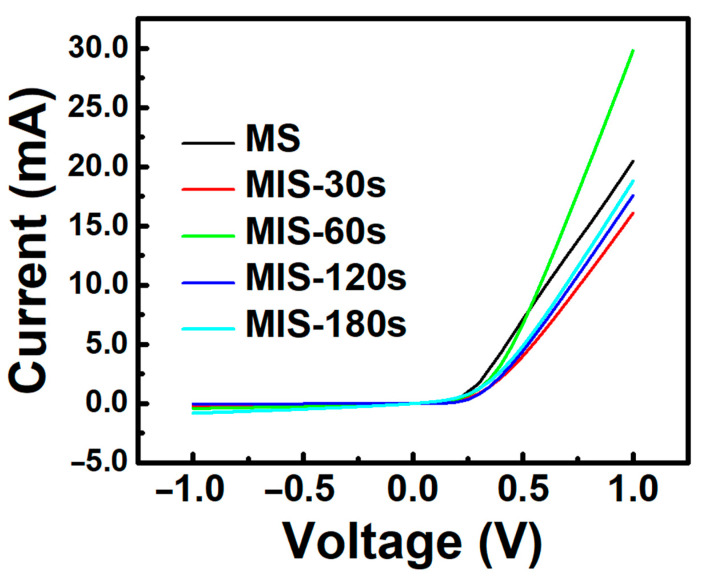
Characteristic curve measurements of Cr/SiO_2_/n-Si devices with different oxidation times.

After optimizing the silicon surface through the oxidation passivation process, the response of the fabricated devices showed an initial increase followed by a decrease as the oxidation time parameter increased. The device with an oxidation time of 60 s exhibited the best response measurement, with a 145% improvement compared to the device without an oxide layer. Additionally, the devices with the oxide layer showed a significant reduction in background noise. In the devices without the oxide layer, the root mean square (RMS) value of the noise was approximately 0.43 nA, which decreased to 0.09 nA after the addition of the oxidation process. This indicates that the presence of an oxide layer has a certain impact on the metal/semiconductor contact interface. To estimate the variation in the barrier height in devices with different process parameters, we also measured and analyzed the I–V characteristics of different devices, as shown in Figure 5. The results of the analysis for R_s_, n, barrier height, and temperature are recorded in Table 3. The barrier height gradually approaches the ideal barrier height (0.45 eV) with the addition of the oxide layer, decreasing from the originally pinned position of 0.56 eV to 0.49 and 0.47 eV. Therefore, in devices with an oxidation time of less than 60 s, the response is optimized with the addition of the oxide layer. Furthermore, from the fitting results of the ideality factor, the ideality factor value continuously increases with the increase in oxidation time. This indicates that the device’s characteristics deviate further from the ideal metal/semiconductor structure. A reasonable ideality factor value generally falls between one and two, and the device with an oxidation time of 60 s already meets the acceptable minimum standard for a diode. A thicker oxide layer would make it difficult for carriers to tunnel from the metal into the semiconductor, resulting in additional decay during measurements. This explains why the response decreases in devices with oxidation times of 120 and 180 s.

**Figure 6:**
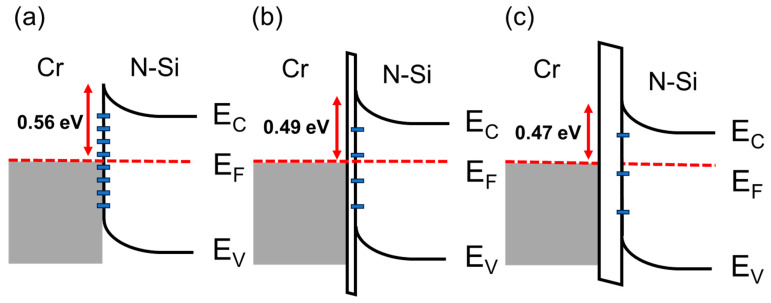
Schematic of energy band diagram of (**a**) Metal-Semiconductor and (**b**) Metal-thin Insulator-Semiconductor and (**c**) Metal-thick Insulator-Semiconductor Schottky interface.

The above discussion addressed the influence of Schottky interface parameters (barrier height, ideality factor) on current. Based on this, we can summarize in a more comprehensive manner how the experimental procedures step by step affect the interface physics and the physical significance represented by the ideality factor. The formation of interface traps occurs on the semiconductor surface. Native defects and local vacancies are often considered as interface traps, which lead to Fermi-level pinning [39,40]. In Figure 6a–c, we illustrate the band diagrams of the metal/semiconductor and metal/oxide/semiconductor structures. When surface defects and traps are present, their energy levels are typically located within the semiconductor bandgap [41]. The density of these traps determines the trapping and releasing processes of electrons and gives rise to the Fermi-level pinning effect. Previous studies on interface trap density at the silicon/silicon dioxide interface have been conducted by several research teams [24,42], indicating that the interface trap density of states decreases with oxide layer thickness, which aligns with the estimations of the Fermi-level pinning effect. Due to the highest density of interface traps, the metal/semiconductor structure in Figure 6a exhibits a pinned barrier of 0.56 eV and noise value of 0.43 nA, and the smallest ideality factor of 1.492. As the oxide structure is introduced, a very thin oxide layer appears at the interface, and the density of interface traps decreases, meaning that the Schottky barrier (0.49 eV) in Figure 6b is between the pinned barrier and the ideal barrier. On the other hand, the insulating layer effectively suppresses dark current (0.09 nA) and enhances device signal performance. However, with the addition of the oxide layer, the structure deviates from the ideal Schottky structure, resulting in an increase in the ideality factor value. When the oxidation time increases further, a thicker oxide layer exists between the metal and the semiconductor, significantly reducing the chance of carrier tunneling, the photocurrent of the interface in Figure 6c also decreases accordingly. At this stage, even though the interface exhibits a barrier height (0.47 eV) closest to the theoretical value, the optical response of this interface exhibits the lowest value.

Detectivity (D*) is widely recognized as the ultimate parameter for evaluating the performance of photodetectors [43,44,45,46]. A crucial aspect of detectivity is that higher values correspond to superior detector performance. It can be defined as the ratio of the signal-to-noise (SNR) generated in a photodetector operating under specific conditions: an incident power of 1 W, an area of 1 cm^2^, and a noise bandwidth of 1 Hz. The utilization of the square root of the area of photodetector in the calculation of detectivity plays a significant role. This factor effectively neutralizes the influence of the size of detector, allowing for a more objective evaluation of the device’s performance. The equation of detectivity is shown below [43]:

(2) $$D^{*}=\sqrt{A\Delta f}\frac{\mathrm{Responsivity}}{I_{\mathrm{Noise}}}$$

where A is the effective area of the detector (6.25 cm^2^) and Δf is the bandwidth. When the Inoise is dominated by the background noise. And the 1 Hz noise spectrum component is taken as the value of the Inoise. Table 4 presents the optical responsivity and detectivity of the MIS-60 s device. The responsivity and the detectivity are obtained by dividing the response currents by the power at each wavelength and Equation (2), respectively. This device exhibits a responsivity in the μA/W range for the 2–6 μm wavelength band, and a detectivity exceeding 10^6^ for the 2–5 μm wavelength band.

## 5. Conclusions

In conclusion, the nanoscale-Cr/n-Si Schottky photodetector has demonstrated that the enhanced responsivity is related to the Fermi-level pinning effect of the Schottky interface. Firstly, by analyzing the I–V curve and the thermionic emission equation of the 10 nm thick Cr/n-Si Schottky device, the Schottky barrier height was determined to be 0.56 eV, which is close to the bandgap of the silicon material. This indicates the presence of a significant Fermi-level pinning effect at the Cr/n-Si interface. Under illumination conditions with a 2 μm light source, the responsivity of the Cr/n-Si device is 167 μA/W. After surface passivation of the silicon material, the initially fixed barrier height gradually decreases to 0.49 eV and 0.47 eV, closer to the theoretical Cr/n-Si barrier height of 0.45 eV. Also, the noise decreases from 0.43 nA to 0.09 nA, and the responsivity exhibits a 45% improvement. Under illumination conditions with a 2 μm light source, the responsivity of the Cr/SiO_2_/n-Si device is 234 μA/W. Furthermore, barrier height engineering allows the devices to exhibit responsivity to light at wavelengths of 4, 5, and 6 μm, which are 5.56, 2.11, and 1.75 μA/W, respectively. Traditionally, the limitation of silicon bandgap has restricted silicon photonics system’s applications in fields such as optical communication and optical sensing. However, sensors with less Fermi-level pinning effect are able to extend the sensing wavelength range, offering the opportunity for silicon-based sensing to operate in a broader spectrum. This advancement can help expand the range and functionality of silicon photonics systems.

## Figures and Tables

**Table 1:** Fitting results of IV curve with external resistance.

Rex (Ω)	0	1	10	100	150
R_s_ (Ω)	29.92	31.00	40.152	131.120	181.033
n	1.492	1.496	1.495	1.498	1.496
Barrier (eV)	0.561	0.566	0.561	0.560	0.561
T (K)	300	298	298	298	298
ΔR_s_ (Ω)		0.981	10.128	101.096	151.009
Relative Error (%)		1.809	1.286	1.096	0.673

**Table 2:** Infrared light response analysis of Cr/SiO_2_/n-Si devices with different oxidation times.

Wavelength (nm)	2000	3000	4000	5000	6000
	Response (nA)				
MS-0 s	14.647	2.932			
MIS-30 s	19.668	2.984			
MIS-60 s	20.557	4.242	0.489	0.186	0.154
MIS-120 s	5.496	1.088			
MIS-180 s	3.772	0.742			

**Table 3:** Analysis of IV curves of Cr/SiO_2_/n-Si devices with different oxidation times.

	MS	MIS-30 s	MIS-60 s	MIS-120 s	MIS-180 s
R_s_ (Ω)	29.924	38.210	20.294	25.194	27.243
n	1.492	1.773	1.97	3.127	3.267
Barrier (eV)	0.561	0.552	0.496	0.495	0.472

**Table 4:** The optical responsivity and detectivity of the MIS-60 s device.

Wavelength (nm)	Responsivity (nA/W)	D* (cmHz^1/2^W^−1^)
2000	2.34 × 10^5^	1.17 × 10^8^
3000	4.82 × 10^4^	2.41 × 10^7^
4000	5.56 × 10^3^	2.78 × 10^6^
5000	2.11 × 10^3^	1.06 × 10^6^
6000	1.75 × 10^3^	8.75 × 10^5^

## Data Availability

The data presented in this study are available on request from the corresponding author. The data are not publicly available due to privacy concerns.
